# Integrated dominance mechanisms regulate reproductive architecture in *Arabidopsis thaliana* and *Brassica napus*

**DOI:** 10.1093/plphys/kiab194

**Published:** 2021-04-29

**Authors:** Catriona H Walker, Cara D Wheeldon, Tom Bennett

**Affiliations:** Faculty of Biological Sciences,School of Biology, University of Leeds, Leeds, LS2 9JT, UK

## Abstract

The production of seed in flowering plants is complicated by the need to first invest in reproductive shoots, inflorescences, flowers, and fruit. Furthermore, in many species, it will be months between plants generating flowers and setting seed. How can plants therefore produce an optimal seed-set relative to environmental resources when the “reproductive architecture” that supports seed-set needs to be elaborated so far in advance? Here, we address this question by investigating the spatio-temporal control of reproductive architecture in Arabidopsis (*Arabidopsis thaliana*) and *Brassica napus*. We show that resource and resource-related signals such as substrate volume play a key role in determining the scale of reproductive effort, and that this is reflected in the earliest events in reproductive development, which broadly predict the subsequent reproductive effort. We show that a series of negative feedbacks both within and between developmental stages prevent plants from over-committing to early stages of development. These feedbacks create a highly plastic, homeostatic system in which additional organs can be produced in the case of reproductive failure elsewhere in the system. We propose that these feedbacks represent an “integrated dominance” mechanism that allows resource use to be correctly sequenced between developmental stages to optimize seed set.

## Teaser

A series of negative feedback mechanisms regulate the spatio-temporal production of reproductive structures in Arabidopsis and *Brassica napus*, allowing plants to optimize seed-set.

## Introduction

In flowering plants, the vegetative phase of the lifecycle is optimized for harvesting resources from the environment; in the shoot system, the primary concern is the capture of photosynthetically active solar radiation. As such, the vegetative shoot architecture (i.e. the spatio-temporal arrangement of organs) of flowering plants tends to consist of a simple, iterative pattern of development. In contrast, the reproductive shoot architecture of flowering plants is optimized to increase reproductive success, utilizing the resources acquired during the vegetative phase. As such, reproductive architecture is driven and constrained by fundamentally different factors to vegetative architecture. In particular, the acquisition of resources, while still beneficial, is less important than servicing the reproductive strategy of the plant. Thus, for insect-pollinated plants where pollinator availability is temporally limited, the need to produce flowers in a single mass display may be particularly important. For other species, the continual initiation of a small number of flowers may be a better strategy. Other plants (such as those growing in the desert) may need to take advantage of a very limited window of environmental opportunity to deposit seeds in the soil. Since flowering plant reproductive strategies are highly diverse, so too are the reproductive architectures through which plants attempt to execute these strategies.

Despite the differences in reproductive architecture between species, the basic building blocks of reproductive architecture are the same among flowering plants. These organs are produced in a hierarchical temporal sequence that is inherently more complex than vegetative architecture. After the plant undergoes the transition to the reproductive phase (i.e. flowering), many or all of the vegetative shoot meristems on the plant will be converted to reproductive shoot meristems (RSMs), which generate reproductive shoots bearing leaves (Benlloch et al., 2015). The secondary “axillary” shoot meristems produced along these reproductive shoots may also be specified as RSMs, but can instead be directly specified as inflorescence meristems (IMs). Existing RSMs may also be converted to IMs during reproductive development. In garden pea (*Pisum sativum*), the main RSMs are long-lived, and axillary meristems are directly specified as IMs (Benlloch et al., 2015). Conversely, in Arabidopsis, every axillary meristem is initially specified as a short-lived RSM, but is quickly converted to an IM after producing a few leaves; this includes the primary RSM (Schultz and Haughn, 1991; Pouteau and Albertini, 2011). IMs initiate bracts (which may be cryptic) rather than leaves, and these bracts contain floral (axillary) meristems. These floral meristems each produce a single flower bearing the male and female reproductive organs (stamens and carpels). Pollination of female gametophytes (the ova, contained within the ovules, within the carpel) by male gametophytes (pollen) leads to the formation of a zygote, and conversion of the ovules into seed. The setting of seed in turn causes the carpel to develop into a fruit, containing the seed. Thus, to produce the seeds that ultimately constitute their reproductive effort, plants must first produce reproductive shoots, inflorescences, flowers, and fruits, in sequence.

The hierarchical and sequential nature of reproductive development makes it difficult for plants to correctly determine their reproductive effort (Walker and Bennett, 2018). The maximum number of seed that a plant can produce reflects the resources it has available at the point of seed set. However, when the plant begins reproductive development, it cannot predict what level of resource will be available at seed set, since this will largely depend on the amount of solar radiation and water the plant receives between the start and end of flowering. This problem is particularly acute in spring-blooming fruit trees, which actually undergo the floral transition the previous autumn, and initiate all the inflorescences and flowers that will “blossom” the following spring. In these species, the initial production of reproductive organs is separated from seed set by up to 9 months, and the resources that may be available for seed set are essentially unknown (Walker and Bennett, 2018). Even in plants with more straightforward reproductive cycles, there may be a large time lag between the initiation of flowering and seed set. How then can plants possibly produce an optimal reproductive architecture, elaborated over weeks, or month of growth, which maximizes reproductive effort? How do plants generate the “correct” number of inflorescences, flowers, fruit, and seed without over- or under-committing resources to any of the developmental stages? Reproductive strategies across species must therefore require control mechanisms to regulate seed set, and adjustment of the reproductive effort may be variously controlled by varying mechanisms, depending on the lifecycle of the plant (Lloyd, 1980).

An important component in structuring reproductive architecture is “correlative inhibition,” in which older reproductive organs inhibit the growth of newer organs (Bangerth, 1989). These phenomena are well known to gardeners and horticulturists, being a prevalent feature of ornamental and horticultural crops. In many species, the earliest fruits will inhibit the development of fertilized and otherwise viable later-set fruit, resulting in reduced growth, abortion, abscission, or senescence (Bangerth, 1989). Cucurbits such as cucumber (*Cucumis sativus*) provide a particularly striking example of this phenomenon, and a single fertile fruit may inhibit any subsequent fruit from developing (Zhang et al., 2015; Shnaider et al., 2018). In many other species, the presence of fertile fruit prevents ongoing flowering, and the prompt “dead-heading” of flowers (in ornamental species) or regular picking of fruit (in horticultural species) is required to promote the continued initiation of inflorescences. A related phenomenon is biennial bearing, in which the presence of a heavy fruit load in spring-blooming trees can inhibit the formation of inflorescences for the next season’s flowering (Samach and Smith, 2013; Krasniqi et al., 2017). In cereal crops, there are well-known “yield trade-offs,” in which increasing one component of yield (e.g. number of ears) will tend to result in the decrease of other components (e.g. seed mass), such that there is no overall increase in the yield (Gaju et al., 2009; Sakuma and Schnurbusch, 2020). Although not formally proven, there is reason to think that these effects are also driven by correlative inhibition mechanisms (Guo and Schnurbusch, 2015).

The traditional explanation for correlative inhibition effects has been competition for resources between organs; that is, plants cannot produce any more organs because the earlier organs monopolize the available supply of resources (Bangerth, 1989). There is certain evidence that resource source–sink relationships within the plant play a role in determining which organs grow and which do not, but also evidence that the effects are not sufficient to fully explain correlative inhibition effects (Marcelis et al., 2004; Wubs et al., 2009, 2011). Furthermore, correlative inhibition of new organs often occurs before they have a high demand for resources (Bohner and Bangerth, 1988). It has therefore been suggested that correlative inhibition occurs by active “dominance” mechanisms, in which older organs inhibit the growth of younger organs by active signaling, rather than by passive resource use (Bangerth, 1989). In the case of apical dominance—the inhibition of new shoot branches by actively growing shoots—the existence of a complex signaling network involving the hormonal signals auxin, cytokinin, and strigolactone has certainly been demonstrated (Domagalska and Leyser, 2011). There is some evidence that the same principles may apply in reproductive development. For instance, auxin transport from early-set tomatoes (*Solanum lycopersicum*) has been implicated in the growth inhibition of later-set tomatoes on the same truss (Bangerth, 1989). In citrus and olive, auxin export from fruit has also been implicated in the inhibition of inflorescence development, and thus biennial bearing (Haim et al., 2021). However, the role of dominance mechanisms in the control of reproductive architecture has not yet been systematically investigated.

Within the Brassicaceae, we now have a very clear understanding of the mechanisms that regulate floral initiation, and the identify and function of shoot meristems during flowering in Arabidopsis (*Arabidopsis thaliana*; Pajoro et al., 2014), and increasingly in other species including *Brassica napus* (O’Neill et al., 2019) and *Arabis alpina* (e.g. Wang et al., 2009; Lazaro et al., 2018; Hyun et al., 2019; Vayssieres et al., 2020). However, despite the prominence of Arabidopsis as a model system, the control of reproductive architecture as a spatio-temporal phenomenon has generally been poorly characterized. Recent work has also started to elucidate how flowering in Arabidopsis is brought to an end, demonstrating the importance of events both in the IM themselves (Wuest et al., 2016; Balanza et al., 2018), and signaling from fertile fruits (Hensel et al., 1994). Indeed, floral arrest in Arabidopsis seems to arise as a result of fruit exerting local dominance over the inflorescence apex by auxin export, although the exact target of this dominance is still uncertain (Gonzalez-Suarez et al., 2020; Ware et al., 2020). However, our understanding of how the number of organs is controlled, and how those are distributed in time and space is lacking. In order to understand the mechanisms that control reproductive architecture in the Brassicaceae, we systematically investigated reproductive architecture control in Arabidopsis, complementing this with targeted experiments in *B. napus*.

## Results

### The scale of reproductive effort is predicted by early developmental decisions

**Figure 1:**
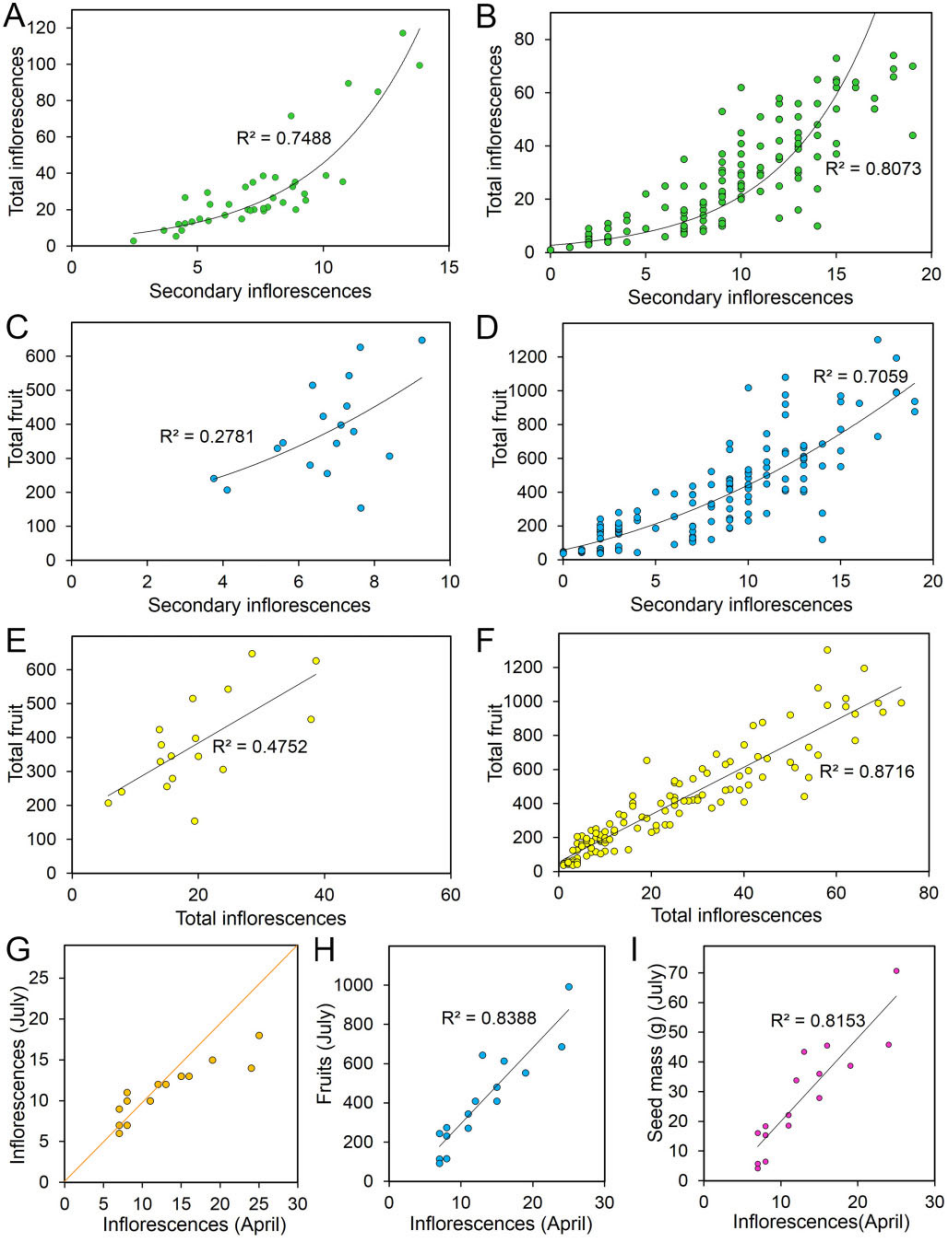
Early events determine the scale of reproductive development. Graphs showing relationship between inflorescence number and reproductive development. Graphs show experimental means for multiple independent studies carried out in Arabidopsis Col-0 (A, C, and E). Plants were grown on compost in either glasshouses or controlled environment chambers, in soil volumes ranging from 50 mL to 2 L. Following the end of flowering, all inflorescences were assessed and recorded, and in some instances, total fruit counts were also obtained (C and E). Graphs B, D, and F show individual plant means for various *B. napus* varieties, from a combination of glasshouse-grown plants in 500 mL or 2 L compost and field-collected samples from commercially grown crops across the UK. Whole plants were harvested from the field, and inflorescence number and total fruit number was assessed and recorded. A and B, Graph showing the relationship between the number of secondary inflorescences and number of total inflorescences in Arabidopsis Col-0 plants (39 sets of experimental means) (A), and in *B. napus* (155 individual plants, various oilseed rape varieties) (B). C and D, Graph showing the relationship between number of secondary inflorescences and number of total fruit in Arabidopsis Col-0 plants (17 sets of experimental means) (C), and in *B. napus* (142 individual plants, various oilseed rape varieties) (D). E and F, Graph showing the relationship between number of total inflorescences and number of total fruit in Arabidopsis Col-0 plants (17 sets of experimental means) (E), and in *B. napus* (132 individual plants, various oilseed rape varieties) (F). Data in (E) have been previously published in Walker and Bennett (2019), but are shown here for the sake of completeness. G, Graph showing the relationship between number of secondary inflorescences in 16 individual commercially grown *B. napus* plants (var. Campus) in April and July. Plants were tagged in April, and inflorescence number recorded. Plants were similarly assessed approximately monthly until the end of flowering (July), at which point plants were harvested and assessed. The orange line indicates the 1:1 line, so dots below the line are plants that lost inflorescences, and above the line gained inflorescences. H and I, Graph showing the relationship between number of secondary inflorescences in 16 individual commercially grown *B. napus* plants (var. Campus) in April and their total fruit number (H) and harvested dry seed mass in grams in July. Plants were tagged in April, and inflorescence number recorded. Plants were similarly assessed approximately monthly until the end of flowering (July), at which point plants were harvested and assessed.

To understand how reproductive architecture is controlled in Arabidopsis, we began by compiling an extensive dataset of reproductive architecture measurements from wild-type Arabidopsis (Col-0) from experiments across the past 18 years. This included experiments grown in a range of growth conditions (glasshouse and walk-in chamber), light intensities, photoperiods, and soil volumes. For each experiment, we had recorded the number of secondary inflorescences, that is, the inflorescences initiated by the axillary meristems in the leaves along the primary shoot axis. This includes both cauline and rosette inflorescence branches (64 experiments; Supplemental Figure S1A). For a number of these experiments, we had also recorded the total number of inflorescences (i.e. including the tertiary inflorescences that branch from the secondaries, the quaternaries that branch from the tertiaries, etc.; 39 experiments; Supplemental Figure S1B), and for a smaller number, the total number of fruits in addition (17 experiments; Supplemental Figure S1C). Because they are easy to count, it is tempting to use the number of secondary inflorescences as a good proxy for reproductive architecture as a whole. Indeed, in studies of shoot branching in Arabidopsis, typically only the secondary inflorescences (usually referred to as “primary branches” in this context) are assessed to define the overall branching levels of plants (e.g. Bennett et al., 2016; Brewer et al., 2016; van Rongen et al., 2019; Fichtner et al., 2020). However, our data show the danger of this approach; while the number of secondary inflorescences does correlate with both overall inflorescence numbers as a whole, the relationship between these parameters is exponential, not linear (Figure 1A). Thus, small increases in the number of secondary inflorescences can produce a dramatic increase in overall inflorescence number—and therefore reproductive effort. Conversely, while plants with more secondary inflorescences certainly tend to produce more fruit, the correlation between these parameters is not strong (Figure 1C). Even total inflorescence number does not strongly predict total fruit number (Figure 1E), as we have previously discussed, because of apparent feedback between active inflorescences (Walker and Bennett, 2019). Nevertheless, it is clear that, as the earliest developmental decision in the elaboration of the reproductive system, the number of secondary inflorescences initiated at the start of flowering broadly determines the overall scale of the reproductive effort in Arabidopsis—especially since Arabidopsis inflorescences have a limited lifetime of activity (Ware et al., 2020).

In a rather smaller dataset of *B. napus* (oilseed rape/canola) plants—including both field and glasshouse grown plants of several varieties—we observed similar patterns. Secondary inflorescence number was correlated with total inflorescence number in a manner also best described by an exponential function (Figure 1B). Interestingly, secondary inflorescence number was much better correlated with total fruit number in *B. napus*, with a quadratic function best describing this relationship; this was also the best fit for the equivalent relationship in Arabidopsis (Figure 1D). Total inflorescence number is exceptionally well correlated with total fruit number, in a clear linear relationship (Figure 1E). In general, *B. napus* tends to produce a smaller proportion of higher order branches (tertiaries and above) than Arabidopsis, which might explain the better correlation between secondary inflorescence number and total fruit number. Although adequately predicting reproductive effort, these inflorescence number parameters nevertheless fail to capture the spatial complexity in the reproductive architecture of *B. napus*, a species in which many flowers do not produce a fertile fruit (discussed further below; Tayo and Morgan, 1975). To understand how early the scale of reproductive architecture is determined in *B. napus*, we tracked the number of active secondary inflorescences in 16 individual field-grown plants from April (before any flowers opened) to July (when plants were nearing fruit ripening). We found that plants with less than 15 secondary inflorescences had essentially the same number in July as in April (Figure 1G), but that those with greater than 15 had all lost inflorescences by July. These were almost all lower-canopy inflorescences; some of these were lost to damage, but the others appeared to have aborted shortly after April. Overall, the inflorescence number in April provided a good prediction of total inflorescence number, total fruit number, and total seed mass in July (Figure 1, H and I). Thus, even more than in Arabidopsis, the overall scale of reproductive development in *B. napus* seems to be determined very early on after flowering; plants produce a reproductive effort completely in proportion to their size in April. Remarkably, larger plants gain no additional advantage from being large, and smaller plants suffer no additional penalty for being small. This is consistent with recent work showing that winter annual *B. napus* plants undergo floral transition, and generate much of their reproductive architecture in autumn, and not spring; their visible flowering thus largely consists of elaborating existing structures, not generation of new ones (O’Neill et al., 2019).

### Resource and resource-related information determine reproductive effort

Our data suggest that the resources available to plants during the vegetative phase, and the resulting pre-flowering developmental history of the plants, may be more important in determining the scale of reproductive effort than the resources available to plants during the reproductive phase. To try and understand which factors influenced the scale of reproductive effort most strongly in our experiments, we interrogated our datasets in more detail. Our fruit number datasets are almost all from plants nominally grown in the same conditions (16-h d lengths, 150–200 µmolm^−2^s^−1^ light intensity, and 100 mL soil volume), so we focused on factors influencing secondary and total inflorescence number. Lighting conditions had relatively little clear effect on reproductive effort, with plants grown in controlled environment rooms versus glasshouses showing the same ranges in inflorescence number. Plants grown in 24 h of light were in the middle of the range of those grown in 16-h d lengths, whereas plants grown in 8-h d lengths were at the upper end of that range (Supplemental Figure S1D). However, it is difficult to disentangle this effect from the altered developmental history of these plants caused by their elongated vegetative phase. It must be noted that we were not able to systematically vary light intensity or quality across experiments to test the effect on reproductive effort, and doubtlessly light availability does play a role in determining this.

**Figure 2:**
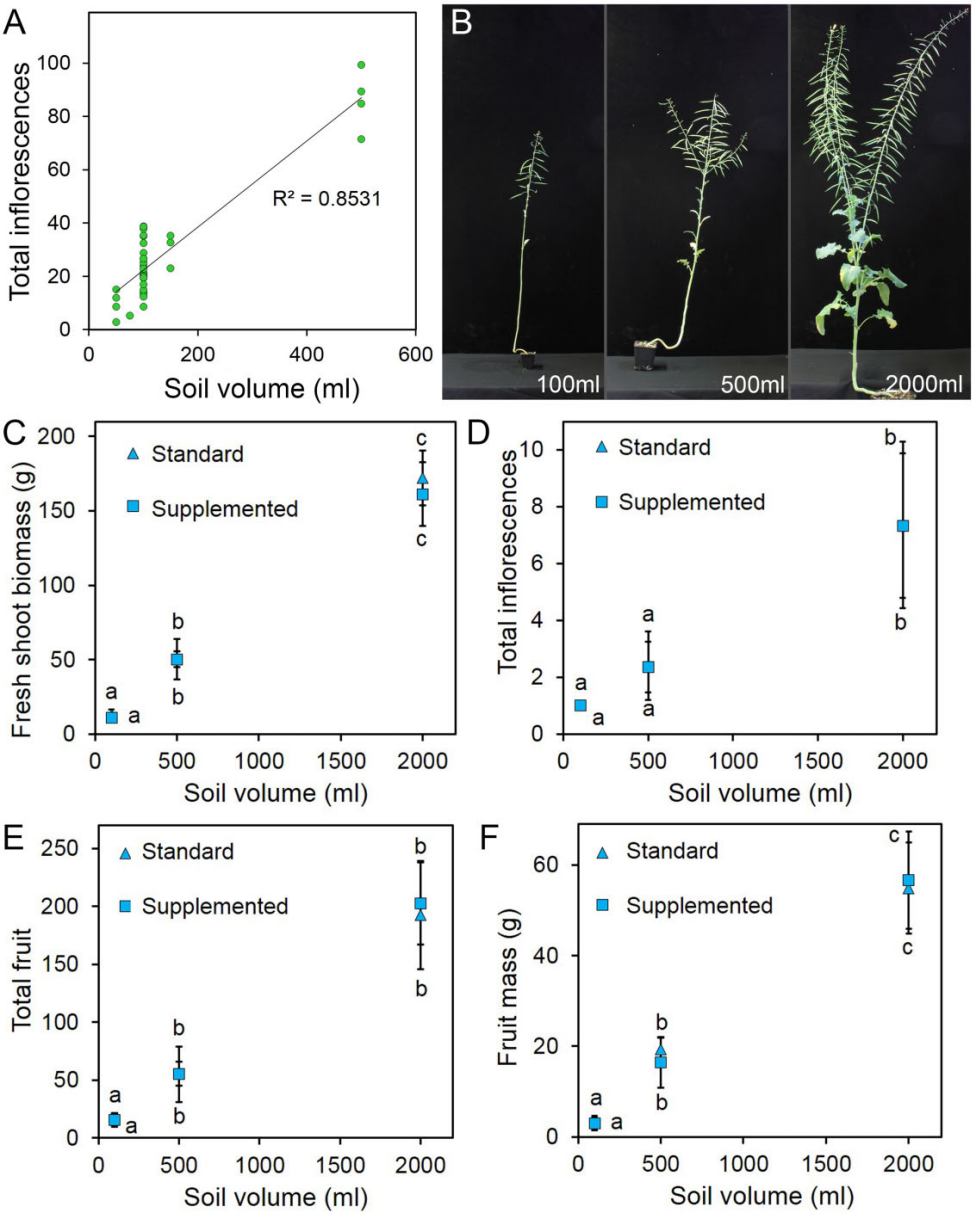
Soil volume directly influences reproductive architecture. A, Graph showing the relationship between soil volume and total inflorescence number in 39 experiments using Arabidopsis Col-0 plants; data points are experimental means. B, Final plant size in Heros spring oilseed rape plants grown in 100, 500, and 2,000 mL of soil (photos are to scale). C–F, Graphs showing the relationship between soil volume and mean fresh shoot biomass in grams (C), mean total inflorescence number (D), mean total fruit number (E), and mean fruit dry biomass in grams (F) in spring oilseed rape grown in 100, 500, and 2,000 mL of soil, with (“supplemented”) or without (“standard”) additional nutrients provided. Error bars indicate sem, *n* = 6–12. Data points with the same letter are not statistically different from each other (C) ANOVA and Tukey’s HSD test, *F* = 374.3; df = 5, *P* > 0.05; (D) Kruskal–Wallis test with pairwise comparison and Bonferroni correction *F* = 59.99, df = 5, *P* > 0.05; (E) Kruskal–Wallis test with pairwise comparison and Bonferroni correction *F* = 62.28; df = 5; (F) ANOVA and Tukey’s HSD test, F = 167.84; df = 5, *P* > 0.05).

The clearest effect on inflorescence number in our dataset appeared to relate to the volume of soil the plants were grown in, with pot size clearly explaining much of the variation in inflorescence number (Figure 2A). This is not especially surprising, since larger pots contain more nitrate (N), phosphate, and other mineral nutrients. However, in many species, substrate volume itself also strongly affects plant growth, independently of nutrient levels (Poorter et al., 2012; Wheeldon et al., 2020). Elsewhere, we have discussed the potential importance of soil volume as a proxy for future resource availability that allows plants to avoid resource limitations (Wheeldon et al., 2020). To test the idea that soil volume is a key determinant of the overall scale of reproductive effort in Arabidopsis, we grew plants in 50, 100, and 500 mL soil volumes. We observed a clear linear relationship between inflorescence number, biomass, and pot size (Supplemental Figure S2, A and B). Additional nutrients had no clear effect on shoot growth parameters in these experiments, suggesting the effect is non-nutritional. To further test whether the effect of soil volume is nutritional or non-nutritional, we grew Arabidopsis in 100 and 500 mL of sand/perlite mix, supplemented with low N, or standard N fertilizer (7.5 or 75 µmol of N/week). Even under low N treatment, plants grown in larger pots had significantly greater shoot biomass than in smaller pots (Supplemental Figure S2E). We observed the same trend under standard N treatment, with plants in larger pots having increased shoot biomass relative to those in smaller pots (Supplemental Figure S2E). Thus, both the volume and nutrient concentration of the substrate affect the growth of the Arabidopsis shoot system. Interestingly, when we grew Arabidopsis in pots larger than 500 mL, we observed a clear saturation of soil volume on reproductive system size. Between 500 and 1,000 mL there was only a marginal and statistically insignificant increase in inflorescence number despite a doubling in soil volume; and similarly between 1,000 and 2,000 mL (Supplemental Figure S2C). We observed the same pattern for shoot biomass (Supplemental Figure S2D). Thus, Arabidopsis growth seems to plateau in the range 500–1,000 mL, above which plants are unable to efficiently use the additional resources available. This might be because of the early flowering in long-day grown Arabidopsis; the plants may not have sufficient time to fully colonize the substrate volume before flowering is induced.

Using *B. napus*, we more clearly defined the effect of substrate volume on reproductive architecture, by growing plants in pots containing 100, 500 or 2,000 mL of soil (Figure 2B). For every parameter we assessed—shoot biomass, inflorescence number, fruit number, and fruit mass—the size of the reproductive system was clearly proportional to the substrate volume (Figure 2, C–F). Again, additional nutrients had no clear effect on shoot growth parameters in these experiments, suggesting the effect of substrate volume is non-nutritional, at least in part (Figure 2, C–F). *Brassica napus* plants grown in 100 mL pots produced only a single primary inflorescence (PI), while Arabidopsis typically produces approximately 30 inflorescences in the same soil volume. Thus, although plants are capable of adapting to any soil volume, the inherent size of the species plays a key role in determining what the reproductive architecture will be in different conditions. In 500 mL pots, the *B. napus* plants produced secondary inflorescences as well, but no tertiaries, while most of the additional branches produced in 2,000 mL pots are tertiaries, rather than secondaries. Thus, like Arabidopsis, the complexity of *B. napus* reproductive architecture increases as overall reproductive effort increases.

### Reproductive shoot/inflorescence branching is homeostatically regulated

We next wanted to understand how Brassicaceae temporally sequence the growth of their reproductive systems in response to resource and resource-related information. In particular, we wanted to understand how plants correctly allocate resources to the early stages of reproductive development. Given that each inflorescence has an essentially fixed growth potential and lifetime (O’Neill et al., 2019; Ware et al., 2020), not making enough inflorescences will restrict maximum reproductive effort. Conversely, since each inflorescence requires an investment of nutrients that cannot be remobilized until the very end of development, and requires a constant supply of water and photosynthate, making too many inflorescences will also restrict reproductive effort. How do plants make the correct developmental “decision” on inflorescence number, such that the resources available for flowering and fruit-set later in development are maximized? We hypothesized that very strong “dominance” effects early on in reproductive development might prevent over-allocation of resources to inflorescence development.

**Figure 3:**
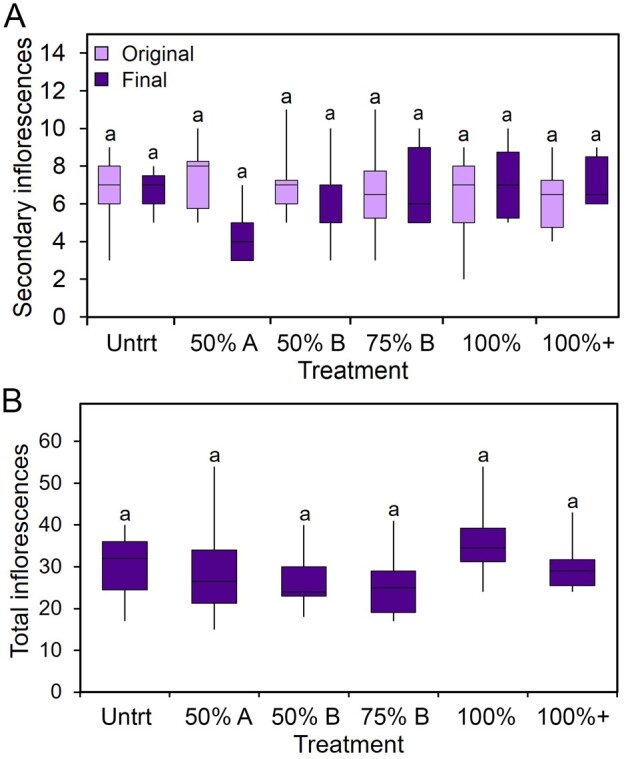
Inflorescence number is homeostatically regulated. A, Graph showing the number of secondary inflorescences produced in Arabidopsis Col-0 plants. At 15 dpb, when the majority of inflorescences had been produced, existing inflorescences were counted (original, light boxes), and scissors were used to remove differing numbers of inflorescences. Then “50% A” had the apical 50% of existing secondary inflorescences removed, while “50% B” and “75% B” had the basal 50% or 75% of secondary inflorescences removed, respectively. “100%” treated plants had all inflorescences removed, while leaving the PI intact, while the PI was also removed in “100% +” treatments. Following recovery and when the plants had finished flowering, the number of secondary inflorescences was again counted (final, dark boxes). Boxes indicate the interquartile range. The central line indicates the median, whiskers show minimum and maximum values. Bars with the same letter are not statistically different from each other (ANOVA + Tukey’s HSD, *n* = 9–12 pre-treatment, 3–6 post-treatment; *F* = 1.165, df = 11, *P* > 0.05). B, Total inflorescences produced following recovery and end of flowering in Arabidopsis Col-0 plants. All treatments as described in (A). Boxes indicate the interquartile range. The central line indicates the median, whiskers show minimum and maximum values. Bars with the same letter are not statistically different from each other (ANOVA + Tukey’s HSD, *n* = 3–6; *F* = 0.737, df = 5, *P* > 0.05).

To test this idea, we performed a variety of inflorescence removal (“debranching”) experiments using wild-type Col-0 Arabidopsis. Although Arabidopsis meristems pass through an exceptionally short-lived RSM phase, by the time any of them are visible (including the primary meristem), they have already converted to IMs. Thus, performing experiments specifically on the reproductive shoot phase of Arabidopsis development is practically impossible, and we treated it as part of the inflorescence phase in our experiments. We trialed different timings for inflorescence removal, before settling on 15 d post-bolting (dpb) as an “early-mid flowering” point. At this point, plants had typically activated most inflorescences, but had only made approximately 30 fruits (∼5% of their typical total fruit set). We performed debranching of secondary inflorescences in different positions, and of different magnitudes. Some plants had 50% of their secondary inflorescences removed, either apically or basally (50% apical and 50% basal), others had 75% removed basally (75% basal); in the most extreme treatments, 100% of secondary inflorescences were removed either without (100%) or in addition to the PI (100%+; Figure 3A). We then tracked the number of secondary inflorescences produced by these plants during the rest of normal reproductive development. Surprisingly, for all treatments, the final number of secondary inflorescences was not statistically different from the original, pre-treatment number (Figure 3A), nor was there any statistical difference relative to the original number of secondary inflorescences present in the untreated control (Figure 3A). The partial exception to this was the 50% apical treatments, which initiated few new secondary inflorescences, although were not statistically different from untreated plants (Figure 3A).

We also tracked the total number of inflorescences produced by these plants. Similarly to secondary inflorescences, there was no statistical difference in the total number of inflorescences between any of the treatment groups and the untreated control at the end of the experiment, including the 50% apical group (Figure 3B). We did not count fruits in this experiment, but in an earlier iteration with the same basic design, we found that final fruit numbers were slightly—but not statistically significantly—lower in the more extreme treatments (Supplemental Figure S3A). We also assessed whether the regulation of inflorescence architecture changes over time by performing the same debranching experiment as in Figure 3A, but in plants which had undergone arrest of the PI (∼22 dpb). We observed highly comparable results to the earlier time points, with the more dramatic treatments resulting in complete replacement of lost organs, while the 50% treatments prompted less strong responses (Supplemental Figure S3B). Thus, as a whole, the Arabidopsis reproductive system displays a very surprising level of homeostasis to loss of organs.

These data demonstrate several key features of Arabidopsis reproductive architecture. First, the initial number of inflorescences produced by the plant does not reflect resource limitations; plants retain the capacity to make the same numbers of inflorescences again, along with attendant fruit, if needed—even if the loss or organs occurs right at the end of reproductive development. Second, the secondary inflorescences collectively inhibit the initiation of new secondary inflorescences, in keeping with the idea of “dominance” between organs. Third, the system is highly homeostatic, and accurately replaces lost organs. In formal terms, the regulatory systems of the plant are calibrated so as to produce an optimal number of inflorescences for the available resources, even if the system is perturbed—but in effect, we could say that the plant has a “target” inflorescence number.

### “Infloretic dominance” arises from all parts of the inflorescence

The clear dominance that secondary inflorescences exert over the activation of other secondary inflorescences is highly reminiscent of the apical dominance phenomenon. However, apical dominance is usually associated with vegetative branching, rather than inflorescence branching. Arabidopsis does not make elongated vegetative branches, and as such has been a poor system for “classical” apical dominance research. Studies using Arabidopsis inflorescences as a model system for apical dominance studies have generally struggled to see the classical effects of decapitation and auxin application (e.g. Chatfield et al., 2000; Cline et al., 2001). However, our results suggest that inflorescences do exert considerable dominance. We therefore questioned whether this “infloretic dominance” is qualitatively different from classic apical dominance in vegetative shoots. We performed experiments in which we removed different parts of the secondary inflorescences to understand how the dominance is mediated. In one treatment, we removed all the flower-bearing parts of each inflorescence, leaving the leaf-bearing nodes at the base of each inflorescence (“de-crowning”). In another treatment group, we removed the IM and cluster of unopened flowers from the inflorescence apex (“de-capitation”), and in another group, we removed all fruit present on each inflorescence (“de-fruiting”). We performed these treatments at 17 dpb, and then tracked the growth of the plants until the end-of-flowering.

**Figure 4:**
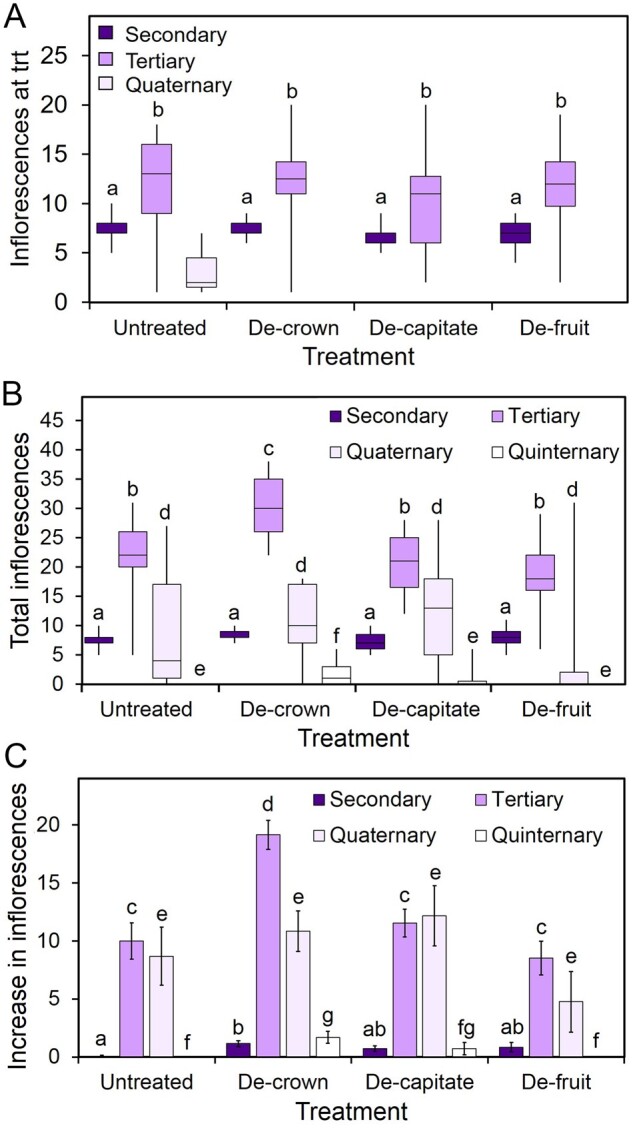
Infloretic dominance arises from a combination of organs. A, Box plot showing the number of inflorescences of each class (secondary, tertiary, and quaternary; dark, medium, and light boxes, respectively) present in Arabidopsis Col-0 plants immediately prior to treatment (trt). When ∼30 flowers had opened on the PI (around 15 dpb), plants were left untreated; had all active floral parts of the inflorescence removed from immediately below the lowest fruit (de-crown); had all bud clusters and IMs removed (de-capitate); or had all present fruit removed (de-fruit). At the time of treatment, the number and position of each inflorescence were recorded. Boxes indicate the interquartile range. The central line indicates the median, whiskers show minimum and maximum values. Bars with the same letter are not statistically different from each other, each class of inflorescence was compared separately (ANOVA + Tukey’s HSD, *n* = 10–12. Secondary *F* = 1.504; df = 3; tertiary *F* = 0.419, df = 3; quaternary *F* = 1.885; df = 3, P > 0.05). B, Box plot showing the number of inflorescences of each class (secondary, tertiary, quaternary, and quinternary; darkest to lightest boxes, respectively) present in Arabidopsis Col-0 plants, following treatment and a recovery period. Treatments were carried out as described in (A). Following recovery and the end of flowering, the total number and position of each inflorescence were recorded. Boxes indicate the interquartile range. The central line indicates the median, whiskers show minimum and maximum values. Bars with the same letter are not statistically different from each other, each class of inflorescence was compared separately (ANOVA + Tukey’s HSD, *n* = 10–12. Secondary *F* = 2.081, df = 3; tertiary *F* = 8.595, df = 3; quaternary *F* = 1.726, df = 3; quinternary *F* = 5.124, df = 3, *P* > 0.05). C, Bar graph showing the difference in secondary, tertiary, quaternary, and quinternary inflorescences between treatment and end-of-life in plants treated as described in (A). Error bars indicate sem; bars with the same letter are not statistically different from each other (comparisons only made within each inflorescence class; ANOVA + Tukey’s HSD, *n* = 10–12. Secondary *F* = 2.891, df = 3; tertiary *F* = 11.951, df = 3; quaternary *F* = 1.830, df = 3; quinternary *F* = 5.124, df = 3, *P* > 0.05).

All three treatments promoted the activation of tertiary, quaternary, and quinternary branches, although inflorescences also increased in untreated plants over the course of the experiment (Figure 4, A–C). This effect was strongest in the de-crowned plants (30 additional branches) and weakest in the de-fruited plants (8 additional branches), which was not statistically different from the untreated group. It should be borne in mind that de-crowned and de-capitated secondary inflorescences will not produce any more organs on existing inflorescences, but those de-fruited inflorescences will continue to produce new flowers and fruits, and therefore represent a much less severe treatment. Intriguingly, we observed that very few new secondary inflorescences were activated in any of the treatment groups (Figure 4, A and B). Thus, although each treatment removed a substantial proportion of the inflorescence, the secondary inflorescences largely retained their dominance over other secondary inflorescences. It therefore appears that the activation of additional tertiary and higher-order branches allows each secondary inflorescence system as a whole to maintain its overall dominance in the system. Thus, the “infloretic dominance” that secondary inflorescences exert over other secondary inflorescences results from the combined dominance exerted by (1) the inflorescence apex, (2) the fruit, and (3) the higher-order branches within the inflorescence system. In this context, it can be noted that while removal of the inflorescence apex (de-capitation) does not have an effect on other secondary inflorescences, it does release higher-order branches subtending the apex from inhibition (Figure 4B). Thus, in the reproductive system, “classic” apical dominance effects only occur within inflorescence systems, and not between them.

### Fruit limit inflorescence activation in *trans* through exchangeable dominance

**Figure 5:**
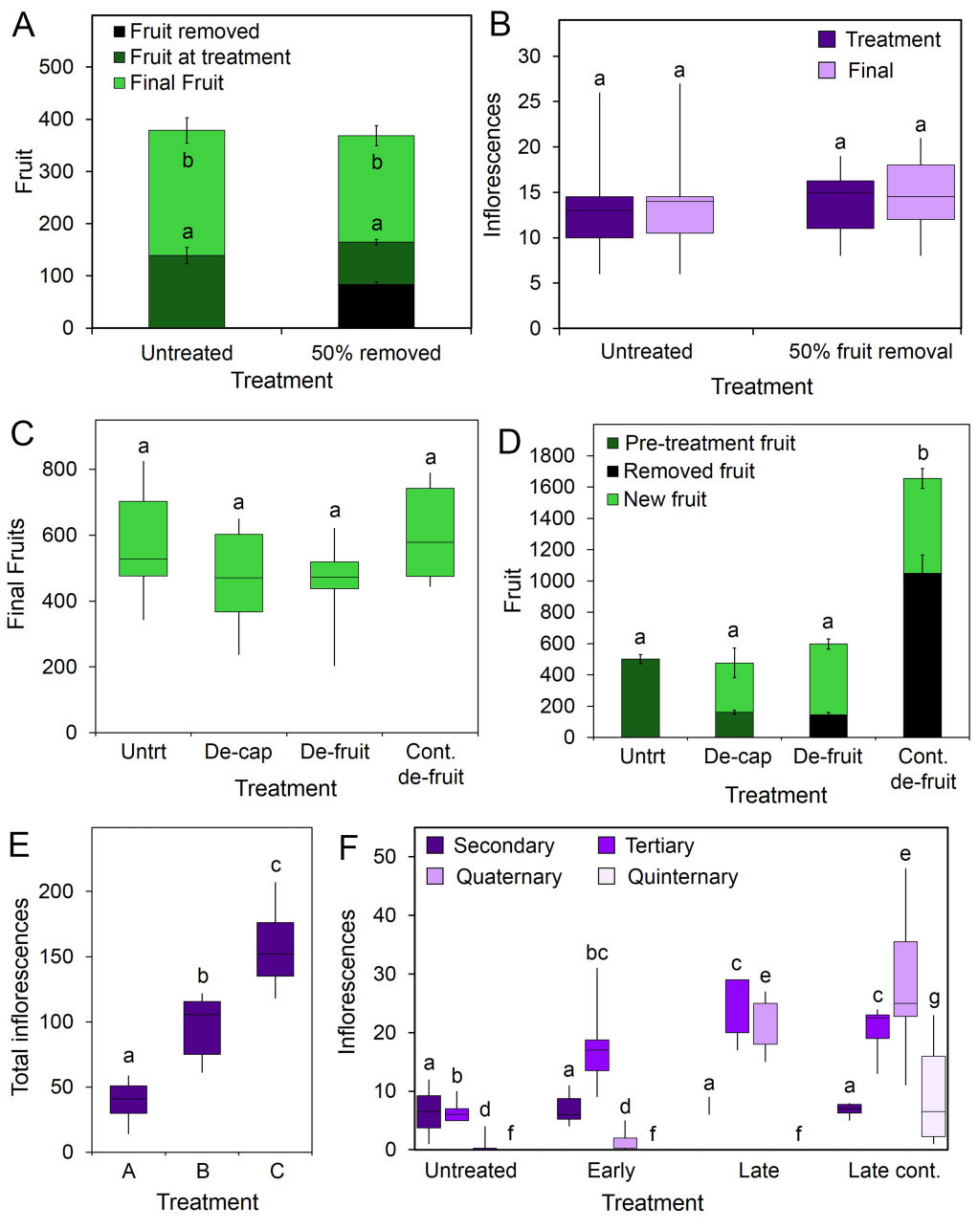
Fruit regulate inflorescence activation in *trans*. A, Graph showing the effects of partial fruit removal on the final total fruit production in Col-0 Arabidopsis. Plants were either untreated, or had the basal 50% of fruit removed from all branches at 17 dpb. The number of fruits removed was counted (fruit removed, black), alongside all remaining fruits on the plant at the time of treatment (fruit at treatment, dark green). Finally, all fruits at the time of floral arrest were counted (final fruit, light green). Bars indicate standard error. Fruit at treatment and final fruit were compared separately; bars with the same letter are not statistically different from each other (T test, *P* < 0.05, *n* = 11–12, *P* > 0.05). B, Boxplot showing the number of inflorescences present following early fruit removal. The basal 50% of fruit was removed from all inflorescences 17 dpb. Inflorescences were counted at the time of treatment (treatment, dark purple), and again following the end of flowering (final, light purple). Box represents interquartile range, and midline indicates the median. Whiskers indicate maximum and minimum. Bars with the same letter are not statistically different from each other (ANOVA + Tukey’s HSD, *n* = 11–12, *P* > 0.05). C, Boxplot showing the final number of fruits produced in Col-0 Arabidopsis following treatment. Treatments were carried out when approximately 30 fruits were present on the PI. At this point, all IMs were removed from the plant (de-cap), all fruits were removed from the plant in a single event (de-fruit), or all fruits were removed, with a period of continual fruit removal, before allowing the plants to recover (cont. de-fruit). Following the end of flowering, the total number of fruits present across the whole plant was recorded. Box represents interquartile range, and midline indicates the median. Whiskers indicate maximum and minimum. Bars with the same letter are not statistically different from each other (ANOVA + Tukey’s HSD, *n* = 6–13; *F* = 2.498, df = 3, *P* > 0.05). D, Graph showing the total number of fruits produced and removed from Col-0 Arabidopsis following treatment. All treatments were as described in (C). Fruits were counted at the time of treatment (pre-treatment fruit, dark green). Fruits removed were counted (removed fruit, black). After the end of flowering, the total number of new fruits produced after treatment was counted (new fruit, light green). Bars indicate standard error. Bars with the same letter do not have a statistically different total fruit number to each other (ANOVA + Tukey’s HSD, *n* = 6–13; *F* = 60.549, df = 3, *P* > 0.05). E, Boxplot showing total final number of inflorescences in Arabidopsis Col-0 plants. Plants were untreated (“A”) or had all open flowers continually removed daily. Plants had all existing fruit and open flowers removed when approximately 30 fruits were present on the PI. Following this treatment, all open flowers were removed daily from every inflorescence for 28 d. After 28 d, the plants were allowed to recover (“B”). The final treatment (“C”) was carried out in the same manner as “B,” only plants were not allowed a recovery period; instead, open flowers were removed daily from these plants until the plants finished flowering. Total inflorescence numbers for each plant were recorded following the end of flowering. Box represents interquartile range, and midline indicates the median. Whiskers indicate maximum and minimum. Bars with the same letter are not statistically different from each other (ANOVA + Tukey’s HSD, *n* = 5–13, *F* = 50.024, df = 3, *P* > 0.05). F, Boxplot showing the effects of severe fruit removal on higher-order inflorescence production. Plants that were “early” treated had all open flowers removed daily from all inflorescences until around 30 fruits (∼15 dpb) were present on the PI, then allowed to flower as normal. “Late” treated plants had all flowers removed daily from all inflorescences, from around 30 fruits being present on the PI; flowers continued to be removed until approximately 30 flowers (∼15 dpb) had been removed from the PI. “Late cont.”-treated plants were as “Late” plants; however, flowers were removed from all inflorescences until approximately 45 flowers had been removed from the PI. At arrest, all present inflorescences within each inflorescence class were counted (secondary, tertiary, quaternary, and qinternary; dark purple to light purple, respectively). Box represents interquartile range, and midline indicates the median. Whiskers indicate maximum and minimum. Bars with the same letter are not statistically different from each other; each class of inflorescence was compared separately (ANOVA + Tukey’s HSD, *n* = 5–11; secondary *F* = 0.741, df = 3; tertiary *F* = 6.131, df = 3; quaternary *F* = 40.097, df = 3; quinternary *F* = 9.292, df = 3, P > 0.05).

These data suggest that fruit plays a role in the control of reproductive architecture of Arabidopsis. To understand the effect of fruit on overall reproductive architecture, we trialed the removal of different numbers of fruit from different inflorescences in Arabidopsis. We found that a wide range of minor perturbations had no effect on reproductive architecture, and that treated plants tended to produce the same number of inflorescences, flowers, and fruit as untreated plants. For instance, plants treated by removal of 50% fruit from the lower part of every inflorescence at 17 dpb made the same total number of flowers and fruit as control plants (Figure 5A). Conversely, more dramatic treatments, such as the removal of all branches (Supplemental Figure S3A), the removal of all fruits (Figure 5, C and D), and the continuous removal of all fruits (Figure 5, C and D) seem to completely “reset” the system, such that treated plants ultimately make approximately the same number of fertile fruits as treated plants. Again, this illustrates that during this later phase of reproductive development, plants retain the capacity to accurately replace lost organs; we could informally say that plants also have a “target” fruit number.

How is this effect of fruit on reproductive architecture mediated? We have previously shown that fruit can limit the further production of fruit on the same inflorescence (i.e. in *cis*) by triggering a time-dependent arrest of the inflorescence (Ware et al., 2020). However, our data suggest that fruits also exert dominance over the activation of higher-order inflorescences (i.e. in *trans*; Figure 4B). To explore this “exchangeable dominance” in more depth, we performed a series of experiments with different fruit removal treatments. Continuous removal of all fruits on the plant leads to a massive increase in the number of branches produced across the plant, while a single complete fruit removal at 17 dpb also induced the activation of a large number of inflorescences (Figure 5E). Compared to these complete de-fruiting treatments, a single treatment removing 50% of the fruit had variable effects. In one experiment, removing the oldest 50% of the fruit on each inflorescence at 17 dpb produced no clear increase in inflorescence number (Figure 5B). However, in other experiments, the same treatment produced quite a strong increase in inflorescence number (Figure 5F), presumably reflecting differences between the developmental stages the plants in different experiments had reached at the 17 dpb. There was also a clear effect of the position of the fruit removed; the removal of the youngest 50% of fruit on an inflorescence had a stronger effect than removing the oldest 50% of fruit (Figure 5F). This likely reflects the timing of the treatments, rather than any major difference in the dominance of the fruits themselves; the first treatment occurring when the IM is still active, and the second after the arrest of the IM (and thereby the end of its dominance from the system). Overall, fruit therefore gradually and collectively supplant the IM as the main source of dominance in the inflorescence, and continue the inhibition of subtending inflorescences. However, the dominance exerted by each Arabidopsis fruit is weak, and it is only once fruit numbers reach their maximum on each inflorescence that they have a strong effect on reproductive architecture.

### Carpic dominance is absent in Arabidopsis

**Figure 6:**
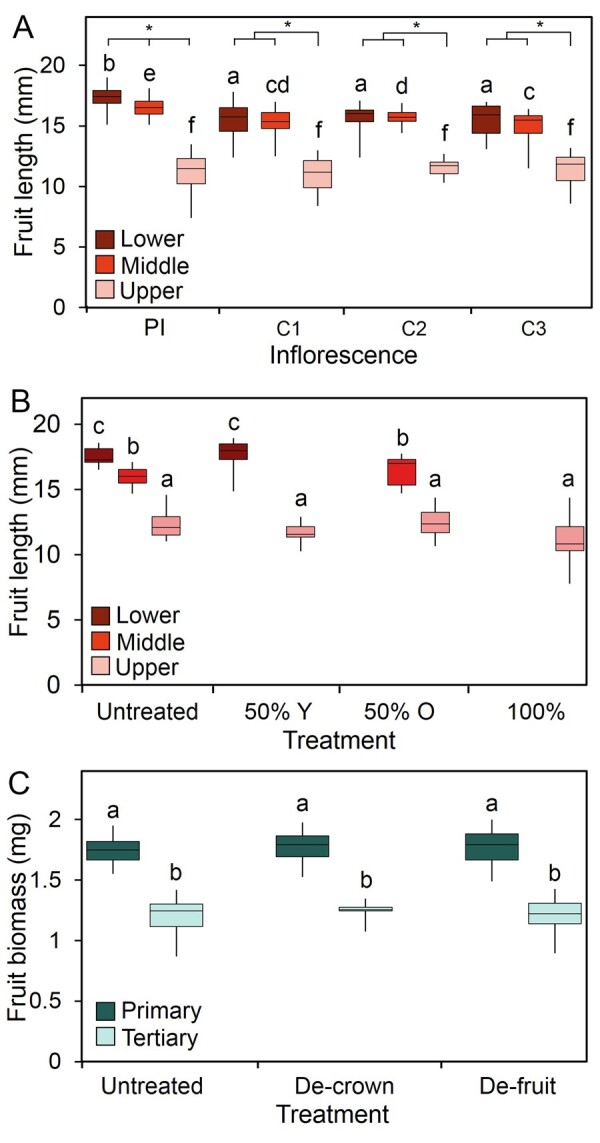
Fruit growth does not show correlative inhibition in Arabidopsis. A, Box plot showing fruit lengths at different positions along inflorescences in Col-0 Arabidopsis. At the end of flowering in untreated plants, fruits were collected from different positions along the PI, and the uppermost three cauline inflorescences (C1–C3, uppermost to lowest). On each inflorescence, three fruits were collected and measured with digital callipers from the lower (dark red), middle (red), or uppermost (light red) part of the inflorescence. These values were then averaged for each section, for each plant. Box represents interquartile range, midline indicates the median. Whiskers indicate maximum and minimum. Bars with the same letter are not statistically different from each other; lower, middle, and upper sections were compared separately across inflorescences (ANOVA + Tukey’s HSD, *n* = 9–12). Asterisks indicated significant differences between sections of the same inflorescence; each inflorescence was compared separately (ANOVA + Tukey’s HSD, *n* = 9–12; lower *F* = 21.318, df = 3; middle *F* = 16.473, df = 3; upper *F* = 1.262, df = 3, *P* > 0.05). B, Box plot showing fruit lengths at different positions along the inflorescence in Col-0 Arabidopsis. When there were approximately 45 fruits on the PI, 50% of the youngest/upper fruit were removed from the PI (50% Y), 50% of the oldest/lowest fruits were removed (50% O), or all fruits present were removed (100%). Plants were allowed to finish flowering, then three fruits were collected and measured with digital callipers from the lower (dark red), middle (red), or uppermost (light red) part of the PI. Values shown are the mean individual fruit length. Box represents interquartile range, and midline indicates the median. Whiskers indicate maximum and minimum. Bars with the same letter are not statistically different from each other; the different sections of the inflorescence were compared separately (upper: ANOVA + Tukey’s HSD, *F* = 2.855, df = 3; middle and lower, *t* test; *n* = 10–11, *P* > 0.05). C, Box plot showing fruit biomass in Col-0 Arabidopsis on primary and tertiary inflorescences. Secondary inflorescences were either “de-crowned,” by having only the flowering part of the inflorescence removed, or had open flowers continually removed (de-fruit). Both treatments were initiated at anthesis of the secondary inflorescence. At the end of flowering, the mean individual fruit biomass for the primary (dark green) and tertiary (light green) inflorescences were calculated. Box represents interquartile range, and midline indicates the median. Whiskers indicate maximum and minimum. Bars with the same letter are not statistically different from each other, inflorescence classes were compared separately (ANOVA + Tukey’s HSD, *n* = 8–12; primary *F* = 0.176, df = 2; tertiary *F* = 0.298, df = 2, *P* > 0.05).

As discussed above, in many species, older fruit inhibit the development of younger fruit on the same inflorescence. The results of this “carpic dominance” may include smaller size of younger fruit (e.g. tomato), shedding of fertile younger fruit by abscission (e.g. apple), or the inhibition or abortion of new fruit development (e.g. cucumber; Bangerth, 1989; Walker and Bennett, 2018). Within the Brassicaceae, the unusual dimorphic fruit of *Aethionema* species have been proposed to arise by carpic dominance (Lenser et al., 2018), but no such phenomena have been demonstrated in Arabidopsis. We therefore examined whether carpic dominance exists in Arabidopsis. We observed that there is a clear gradient in fruit size along each inflorescence with smaller fruit toward the apex; and also between inflorescences, with higher-order inflorescences having smaller fruit than major inflorescences (Figure 6A). This developmental gradient is suggestive of carpic dominance, so we tested this by removing either the 50% oldest, the 50% youngest, or 100% of fruit from the PI at ∼20 dpb, and assessed the effect on the growth of the subsequent fruit on the inflorescence. However, we observed no change in the size of post-treatment fruit, even in the strongest treatments, strongly suggesting there is no same-inflorescence carpic dominance in Arabidopsis (Figure 6B).

We reasoned that carpic dominance might still occur in Arabidopsis if fruit on higher-order branches are inhibited by fruit on the super-tending branch. We, therefore, removed the fruit from all secondary inflorescences, to test whether this had any effect on the size of fruit on the tertiary inflorescences. Since we have already shown the infloretic dominance of major inflorescences arises from a combination of fruit and IM, we also performed a de-crowning of the secondary inflorescences, to test whether this altered fruit size on tertiary branches. However, neither treatment had any effect on fruit size (Figure 6C). We, therefore, conclude that in Arabidopsis, there is no detectable correlative inhibition of fruit by any organ type.

### Older fruit cause the abortion of younger fruit in *B. napus*

**Figure 7:**
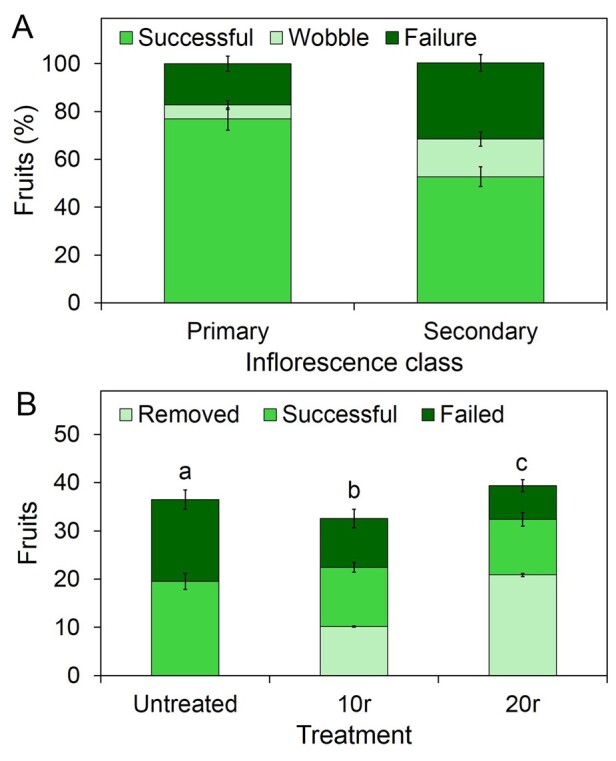
Carpic dominance effects in *B. napus*. A, Graph showing the percentage of fruit “zones” along inflorescences of different classes in untreated *B napus*. Plants were grown in 2,000 mL pots in the glasshouse under supplemental light conditions. At the end of flowering, each fruit on each inflorescence was assessed as “successful” (fruit containing seeds) or “failed” (a produced flower which resulted in no seeds). Percentage “zones” of success were then determined—the successful (green) zone encompassed the lowest fruit, to the highest successful fruit, where no failed fruits were present. The “wobble” zone encompassed the zone in which both successful and failed fruits were present. The failure zone encompassed the uppermost portion of the inflorescence where no successful fruits were present. Bars indicate sem *n* = 6. B, Graph showing number and success of fruit in *B. napus* under different treatments. Plants were grown in 500 mL pots in the glasshouse under supplemented light conditions. Plants were either left untreated or had the first 10 or 20 open flowers on the PI removed before pollination (10 and 20r (removed), respectively). All plants were then allowed to finish flowering, at which point the fruits on the PI were counted and assessed. Fruits were scored as either removed (light green), successful (a fully formed fruit containing seeds) (green) or failed (any flower produced which did not produce seed) (dark green). Bars represent sem. Bars with the same letter indicate plants where the number of failed fruits was not significantly different (ANOVA + Tukey’s HSD, *n* = 7–9; *F* = 18.518, df = 2, *P* > 0.05).

*Brassica napus* has previously been suggested to show carpic dominance (Bangerth, 1989), since fruit development is typically inhibited toward the end of inflorescences lifetimes, such that the final period of flowering in oilseed rape may not produce any fertile fruit (Tayo and Morgan, 1975). We characterized the extent and occurrence of this phenotype in our growth conditions, in plants grown in 2,000 mL of soil. Under these conditions, we observed that the fruit-set is generally highly successful early on in development, but begins to decline after (on average) 72% of flowers have opened. There is a short “wobble zone” with a mixture of fertile and aborted fruit, and then the final 20% (on average) of flowers generally produce no fruit (Figure 7A). The same pattern is seen on the secondary inflorescences, but the “zone of success” is always proportionally shorter than on the PI—and in late activating secondary inflorescences, as few as 40% of flowers may result in a fertile fruit (Figure 7A). This pattern of development is thus highly consistent with carpic dominance effects, but as we saw in Arabidopsis, does not necessarily arise by correlative inhibition.

We therefore tested whether older fruits do indeed inhibit the formation of fruit in the later flowers on the inflorescence, by removing either the first 10, or first 20 flowers produced along the PIs of oilseed rape plants grown in 100 mL of soil. Plants grown in this soil volume typically only produce a single inflorescence, removing any confounding effects of other inflorescences in this experiment. Under these conditions, 46% of the flowers in untreated plants did not lead to production of a fertile fruit (Figure 7B). However, when the first 10 or 20 flowers are removed, was a strong reduction in the “failure” of subsequent flowers to produce a fertile fruit (Figure 7B). Thus, although there is no evidence for carpic dominance in Arabidopsis, this phenomenon does seem to occur in *B. napus*.

## Discussion

### The control of inflorescence number and development

In this study, we set out to understand the mechanisms that shape the spatio-temporal organization of reproductive organs in Arabidopsis and *B. napus*. The earliest visible events during the reproductive development of both species are the activation of secondary inflorescences, which occur immediately after floral transition. Once active, the number of secondary inflorescences remains relatively constant in both species during reproductive development (Figures 1G and 3B), and broadly predicts the overall scale of the reproductive effort (Figure 1, D, G, and H). Our results indicate that secondary inflorescence number is very tightly controlled, and that we can perhaps speak of plants having a “target inflorescence number.” When the system is pushed away from this number, the plant responds by initiating new secondary inflorescences until the original target is reached again (Figure 3A). This shows that inflorescence number is controlled by the concerted dominance exerted by the secondary inflorescences over other secondary axillary meristems. Our results show that this “infloretic dominance” is a property of the whole secondary inflorescence system; the IM, fruit, and subtending tertiary branches (Figure 4, A and B). Removal of the fruit or meristem does not remove the dominance of the secondary inflorescence as a whole, but rather allows the activation of more tertiary branches, which maintain the overall dominance of the secondary inflorescence system. Our results thus show that higher-order branches are regulated by both the IMs and fruits. As fruit numbers increase, and the IM gradually shuts down, there is seamless “exchangeable” dominance that continues to inhibit higher-order branches. Our results stress the importance of fruit in the control of further inflorescence formation; reproductive success, therefore, tends to limit further flowering, while reproductive failure promotes its continuation. The importance of dead-heading and/or prompt fruit-picking to maintain flowering illustrates that fruit also play a key role in preventing the activation of new inflorescences in many other species beyond the Brassicaceae. Indeed, heavy fruiting is even able to inhibit the formation of inflorescences for the next year’s flowering, generating the biennial bearing habit seen in many fruit trees (Krasniqi et al., 2017).

### The control of fruit number and development

In many species, fruits have been demonstrated to exert carpic dominance over the growth of other fruit (Bangerth, 1989). Our results clearly demonstrate that fruits exert dominance over inflorescences in Arabidopsis, but show that fruits exert no dominance over other fruit. This situation seems somewhat paradoxical, especially since *B. napus* seems to display carpic dominance, as do members of the Brassicaceae genus *Aethionema*, which have dimorphic fruits (Lenser et al., 2018). However, it is worth noting that in Arabidopsis, fruits do exert dominance over the continued opening of flowers on the same inflorescence (i.e. in *cis*; Ware et al., 2020). Could it be the case that in Arabidopsis, carpic dominance effects are actually so strong that they act at a much earlier stage of development, and inhibit flowers from ever opening, rather than inhibiting the fruit set of opened flowers? From a different perspective, we might also question why there is this discrepancy in reproductive strategy between Arabidopsis and *B. napus*. Why does *B. napus*—and many other species besides—abort or otherwise inhibit the growth of viable fruit? One possible explanation is the pollination strategy of different organisms. Arabidopsis is highly self-fertile, to the point where it pollinates the majority of its own flowers before they open. For Arabidopsis, production of a flower essentially guarantees production of a fruit, and fruit number can be controlled as function of flower number. However, for insect-pollinated *B. napus*, opening a flower does not necessarily guarantee a fruit will be produced, and the plant may need to “over-flower” to produce the required fruit set. In turn, this requires the plant to have a carpic dominance mechanism to prevent excess fruit set if pollination is more successful. The need for such a system may be particularly strong in spring-blooming fruit trees such as apple, where the inflorescences are all formed the previous autumn. Because pollinator availability in spring is unknown, the plant must produce many more flowers than needed to ensure a minimum fruit-set. In the event of good pollinator availability, excess fruit are removed in the remarkable “June drop” (Abruzzese et al., 1995).

### An integrated model for control of reproductive architecture in Brassicaceae

**Figure 8:**
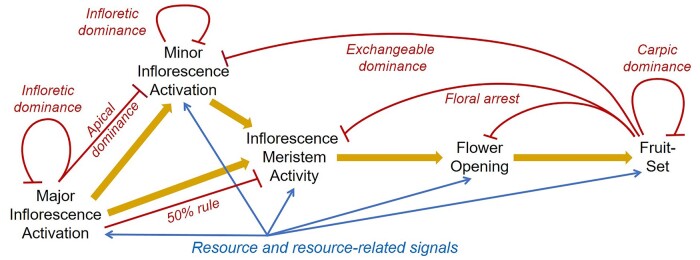
A model for spatio-temporal control of reproductive architecture. Diagram showing developmental processes in the elaboration of reproductive architecture in the Brassicaceae, and their interrelationship (gold arrows). Negative feedbacks identified in this or other studies are indicated with red arrows (50% rule: Walker and Bennett, 2019; Floral arrest: Ware et al., 2020). Positive feedback from resource or resource-related signals is shown in blue.

Taken together, our results suggest that there is an integrated dominance mechanism that acts throughout reproductive development in Arabidopsis and other Brassicaceae, to coordinate the growth of reproductive organs in space and time. The source and target of this central dominance mechanism may change during development, but the transitions between are relatively “seamless.” We can nevertheless identify different dominance interactions that occur at different stages in reproductive development (Figure 8). Within this system, resources and resource-related signals such as substrate volume seem to be the main determinant of how many organs can form in total. The dominance mechanisms then determine how this growth potential is divided among different classes of organs, to determine which organs grow. Each secondary branching system shares a proportion of the overall growth potential, and this is distributed (and homeostatically re-distributed) within the branching system. Higher-order inflorescences have an inherently lower growth potential than secondary inflorescences, and only grow if there is “spare” growth potential - if resource availability is high, or if the secondary “crown” is damaged. In *B. napus*, the lower growth potential of higher-order branches is also reflected in the much lower proportion of flowers that set a fruit, showing that the hierarchical position of an inflorescence affects more than just its growth. This integrated dominance mechanism generates a flexible, homeostatic system, allowing more organs to be produced either locally or globally, depending on changes in environmental conditions, and depending on the earlier reproductive success of the plant.

Here, we have not attempted to elucidate the molecular regulation underpinning the dominance network. However, it is very likely that a combination of hormonal signals—and in particular, auxin, cytokinins, and strigolactones—form the core of this system. An extensive body of work has identified these hormones as critical regulators of the apical dominance that occurs in vegetative shoots, in which actively growing apices repress the activation of new shoot branches (Domagalska and Leyser, 2011). Auxin exported by dominant shoot apices seems to act by occupying the auxin sink strength of the stem, which prevents dominated apices from forming a canalized auxin transport link to the stem, and from exporting their own auxin (Prusinkiewicz et al., 2009; Shinohara et al., 2013; Bennett et al., 2016; van Rongen et al., 2019). Meanwhile, cytokinins and strigolactones, respectively, promote and repress the activation of new branches by increasing or decreasing the abundance of PIN auxin transporters in the stem, thereby altering auxin sink strength (Shinohara et al., 2013; Waldie and Leyser, 2018) and by priming or de-priming apices for growth (Dun et al., 2012). There is reasonable evidence that the dominance mechanism(s) active during reproductive development operate on the same principles, or are indeed the same mechanism. For instance, the *cis*-effect of fruit on inflorescence activity in Arabidopsis is driven by auxin export from fertile fruit (Ware et al., 2020), and the biennial bearing effect of citrus and olive fruits is mediated by increased auxin transport from the fruits (Haim et al., 2021). Meanwhile, in *Aethionema arabicum*, cytokinin treatment increases the proportion of large “dominant” fruit relative to the small “dominated” fruit (Lenser et al., 2018). We thus believe that, as indeed previously proposed by Bangerth (1989), reproductive organs must export auxin in order to grow, and can be inhibited from doing so by the auxin export from actively growing organs—whether of the same type, or different. This model requires further investigation, but provides a preliminary framework for the control of reproductive architecture in Brassicaceae.

### Early, resource-related developmental decisions shape reproductive architecture

For a sustainable future, crop yields must be increased without using additional land for agriculture, and indeed with reduced inputs of fertilizer, agrochemicals, and oil-driven machinery. In other words, there is a pressing need to “do more with less.” There is certainly scope to do this, given that the yields of most crop plants are generally well below the yields that are theoretically achievable given the water, sunlight, and mineral nutrients available to them (Foulkes et al., 2009; Mitchell and Sheehy, 2018; Schills et al., 2018). We therefore need to understand the constraints that prevent plants from achieving such yields. Our results suggest that the scale of reproductive development is largely established very early on during the reproductive process, probably reflecting environmental conditions and developmental events during the vegetative phase. While both species can flexibly respond to environmental conditions post-flowering by making more or fewer higher order inflorescences, these are rather unproductive in *B. napus* (Figure 7A), and produce smaller fruit in Arabidopsis (Figure 6A), and do not dramatically increase the overall reproductive effort. It is also notable that, at least in the case of nutrients, post-flowering increases in availability had very little effect on any aspect of reproductive development in our experiments (Figure 2). Our data suggest that—from a structural perspective at least—it is critical to increase the production of major inflorescences (e.g. ears in wheat, secondary inflorescences in *B. napus*), at the very start of flowering to achieve dramatic increases in yield potential of crops. However, how can this be successfully achieved in practice? Our results, along with those of others, show that simply increasing the quantity of secondary inflorescences will not necessarily increase yield, due to the homeostatic feedback in the system (Walker and Bennett, 2019).

Our data suggest that one way to achieving this increase may be to alter the way that plants respond to resource and resource-related signals, which strongly determine the overall size of the reproductive system. In particular, we show that the substrate volume in which plants are growing strongly limits the scale of their reproductive effort, independently of the mineral nutrients available in the substrate (Figure 2; Supplemental Figure S2A), consistent with our previous work in wheat (Wheeldon et al., 2020). Although substrate volume may seem like an abstract concept for field-grown plants, our results suggest that substrate volume and neighbor density are at least partly interchangeable, and that small pots effectively mimic high neighbor density (Wheeldon et al., 2020). Furthermore, substrate volume effects could arise under field conditions from shallow soil or compacted soil layers. Our results suggest plants may be inherently “cautious” about reproductive development when substrate volume/neighbor density indicates there may be future resource limitations, and do not maximize their reproductive potential relative to the actual abundance of resources. Indeed, as we have previously discussed, this non-maximization of reproduction is a very sound strategy for wild plants (Walker and Bennett, 2018), but is maladaptive in crops where human intervention guarantees future resource availability. Thus, by changing the way plants respond to resource-related signals, there seems to be scope to increase the scale of the reproductive effort, and ultimately crop yield potential.

## Materials and methods

### Plant growth conditions and materials

Arabidopsis (*A. thaliana*) plants for the experiment described in all figures were grown on a Levington’s F2 or Petersfield No. 2 compost, or in a 1:1 sand/vermiculite mix under a standard 16-h/8-h light/dark cycle (20°C/16°C), primarily in controlled environment rooms with light provided by white fluorescent tubes at intensities of ∼120 μmol/m^2^s^−1^, unless otherwise specified. Oilseed rapes were grown on Petersfield No. 2 compost in greenhouses with supplemental LED sodium lighting to an average intensity of ∼250 μmol/m^2^s^−1^. The lines used in this study were wild-type Col-0 (Arabidopsis), and spring oilseed rape variety Heros (*B. napus*).

We used *A. Thaliana* Salts (ATSs; Wilson et al., 1990) as a standard modular fertilizer, and we varied the nitrate concentration by replacing nitrate ions with chloride. Standard N fertilizer was 0.015 M nitrate, low N fertilizer was 0.0015 M nitrate. Plants grown on sand/vermiculite received 5 mL of ATS + 5 mL water once per week in place of watering. Plants grown on compost received 5 mL of standard ATS + 5 mL of water (Arabidopsis) or 10 mL of standard ATS (*B. napus*) every week in place of watering.

### Sampling of field grown plants

For Figure 1, B, D, and F, *B. napus* oilseed rape plants (various varieties) grown at a variety of sites in the UK were hand-harvested at the end of their life, and measured in lab conditions. For Figure 1, G–I, 16 plants in commercial cultivation at the University of Leeds farm were randomly selected in March and marked with tape, and a GPS location. We returned to measure these same plants in situ in April, May, June and July. The mature plants were hand-harvested in July and returned to the lab for final measurements. Fruit were collected and dried, and their biomass measured. Seed were subsequently harvested from the fruit and their biomass was measured separately.

### Inflorescence nomenclature

Inflorescences are referred to typically through their positions and orders. The PI is the main inflorescence growing first, directly from the center of the rosette. The inflorescences which arise from the cauline leaves on the PI are referred to as secondary inflorescences, or cauline inflorescences. Inflorescences arising directly from the rosette leaves (but which are not the PI) are also classed as secondary inflorescences, and are referred to as rosette inflorescences. Any inflorescence which grows from a secondary inflorescence, regardless of whether it is a cauline or rosette, is referred to as a tertiary. Correspondingly, tertiaries give rise to quaternary inflorescences, which in some cases also produce quinternary inflorescences (Supplemental Figure S4).

The inflorescence initiation in Arabidopsis is basipetal (from top to bottom), with the oldest inflorescences being the caulines, with the rosettes initiating thereafter. As inflorescences grow upwards, the youngest part of the inflorescence is the top, with the oldest being the bottom.

The nomenclature used here also applies to *B. napus*. The overall growth of the two species is highly similar, only differing in that *B. napus* produces no rosette inflorescences; the descriptions are otherwise the same between both. To allow for greater clarity when making comparisons, we also refer to “fruits” throughout the manuscript; these are commonly referred to as siliques in Arabidopsis and pods in *B. napus*; however, they both fit the broader classification of fruit.

### Experimental design

#### Soil volume experiments

To determine the effects of soil volume on reproductive architecture in *B. napus*, plants were grown in compost in three pot sizes; 100, 500, and 2,000 mL. From 3-week old, supplemental fertilizer was provided to half of the plants of each pot size weekly, in the form of 10 mL standard*Arabidopsis thaliana* Salts (ATS) media, following regular watering. Standard and supplemental plants were kept in separate trays to ensure any run off could not be accessed accidently by a plant undergoing different treatment. Different pot sizes were similarly kept in different trays to eliminate any effects of shading by larger plants. Plants were grown until the end of flowering, and the development of the final fruit. At this point, all inflorescences were recorded and fruits were harvested from the plant, with fruit number per inflorescence recorded, and biomass measurements were taken of the whole fruit mass per plant. All fresh shoot biomass above the surface of the compost was harvested and biomass measurements taken separately (Figure 2, B–G; Supplemental Figure S2).

To determine the effects of root restriction and nutrition in Arabidopsis (Supplemental Figure S2, A and B), plants were grown in compost in three pot sizes; 50, 100, and 500 mL. Standard plants received no additional fertilizer, while supplemental plants received 5 mL standard ATS + 5 mL water weekly in place of watering from 1 week old. Plants were grown until the end of flowering, at which point all inflorescences were recorded. The shoot was cut immediately above the rosette leaves and dried in a drying oven. Biomass was recorded using an electronic balance.

To test the uppermost limits of Arabidopsis growth (Supplemental Figure S2, C and D), plants were grown as above, only in compost volumes of 100, 500, 1,000, and 2,000 mL with no supplemental fertilizer. Measurements were collected as above.

To assess the effects of fertilizer restriction on Arabidopsis shoot biomass (Supplemental Figure S2E), plants were grown on a 50:50 sand:vermiculite mix, with a small (∼0.5 cm^3^) compost plug to enable germination and establishment. Plants were grown in 100 or 500 mL pots, with 5 mL fertilizer (low N or high N) + 5 mL water applied weekly in place of watering from 1 week after sowing. Plants were grown to the end of flowering, and shoot biomass was collected as described above.

#### Inflorescence and fruit manipulation experiments

For Arabidopsis experiments where inflorescence manipulations were carried out, plants were grown in 50 mL compost with 24 plants per tray. Treatments were randomized across all trays using a random number generator at the beginning of the experiment. Floral transition timings (bolting, the first day of visible buds within the rosette) were recorded for each plant to ensure treatment timings were carried out at the correct time (typically 15 dpb, specified in the text where different; Figures 3–7).

To determine the correct inflorescences were surgically removed, all inflorescences were counted at the time of treatment. Where exact inflorescence removal could not be carried out (e.g. when the treatment called for 50% removal of an odd number of inflorescences), the effects of architecture were considered. About 50% inflorescence removal typically resulted in removing all the cauline or all the rosette inflorescences—when an odd number of inflorescences were encountered, removal was generally kept to either cauline or rosette where possible.

Inflorescence removal was carried out using scissors to remove the entire inflorescence and all subtending higher-order branches, by removing the inflorescence ∼1 cm from its base. De-crowning treatments differed in that they involved only the removal of the flower-bearing section of the inflorescence, leaving any subtending inflorescences and buds intact. The inflorescence was removed with scissors ∼5 mm below the lowest fruit on that inflorescence. De-capitation treatments involved using forceps to remove the bud cluster and IM from a treated inflorescence. The bud cluster was removed above the uppermost open flower. De-fruit treatments involved using scissors or forceps to remove every fruit and open flower. For treatments where continual flower removal was carried out, this was carried out daily on all inflorescences present, unless otherwise specified.

#### Fruit measurements

Fruits in Arabidopsis and *B. napus* were measured in the same way. Ripe fruits were removed from the plant and length was measured from where the pedicel meets the fruit to the fruit tip, using digital callipers. For biomass measurements in Arabidopsis, three fruits were grouped together and weighed using an electronic balance, before a mean individual fruit mass was calculated (Figures 6 and 7).

For all fruit measurements, fruits were collected when ripe; in some instances (in Arabidopsis only) this meant collecting groups of fruits from the plant at different timings to ensure all growth had finished, but seeds were not lost. When total shoot biomass was collected, all biomass above the surface of the growth medium was harvested and biomass was added to the fruit mass.

## Supplemental data

The following materials are available in the online version of this article.

**Supplemental Figure S1.** Early decisions shape reproductive architecture in *B. napus*.

**Supplemental Figure S2.** Substrate volume determines shoot growth in Arabidopsis.

**Supplemental Figure S3.** Inflorescence and fruit number display homeostasis in Arabidopsis.

**Supplemental Figure S4.** Inflorescence nomenclature in Arabidopsis and *B. napus.*

## Supplementary Material

kiab194_Supplementary_Data

## Notes

C.H.W., C.D.W., and T.B. designed and performed experiments and analyzed the data. T.B. and C.H.W. conceived the project. All authors contributed to writing the manuscript.

The author responsible for distribution of materials integral to the findings presented in this article in accordance with the policy described in the Instructions for Authors (https://academic.oup.com/plphys/pages/general-instructions) is: Tom Bennett (t.a.bennett@leeds.ac.uk).
