# Supplementary material for: Integrated dominance mechanisms regulate reproductive architecture in Arabidopsis thaliana and Brassica napus
Source: Plant Physiol. 2021 Apr 29;186(4):1985–2002. doi: 10.1093/plphys/kiab194 (PMC8331136; doi:10.1093/plphys/kiab194)
Supplement: kiab194_Supplementary_Data [file kiab194_supplementary_data.pdf]

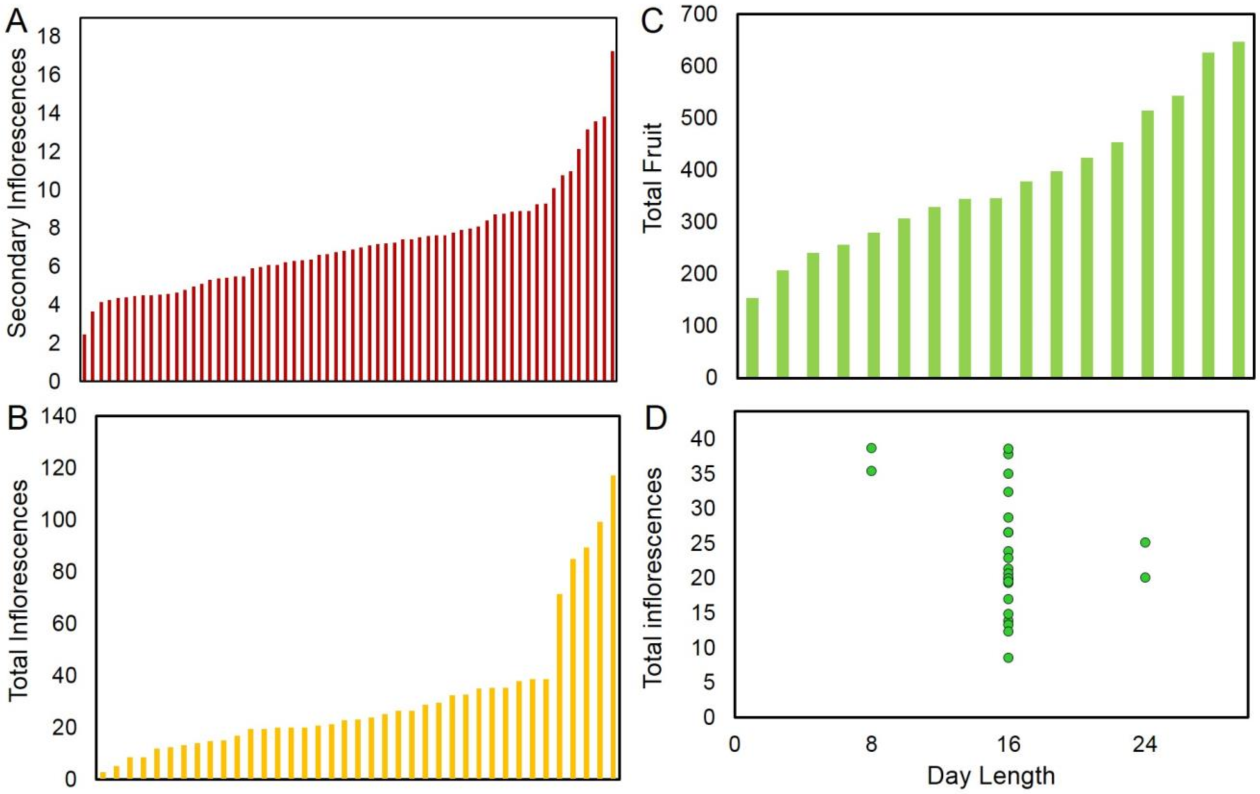

Supplemental Figure S1: Arabidopsis reproductive architecture datasets

A-C) Graphs showing the ranked experimental means for the number of secondary inflorescences (n=64 experiments)(A), total inflorescences (n=39)(B) and total fruit (n=17)(C), from a series of experiments performed with Arabidopsis Col-0 wild-type plants, from 2002-2020, using a variety of soil volumes and growth conditions.

D) Graph showing total inflorescences produced in Arabidopsis Col-0 in 25 experiments conducted in different day-lengths. Each data point is the mean of one experiment.

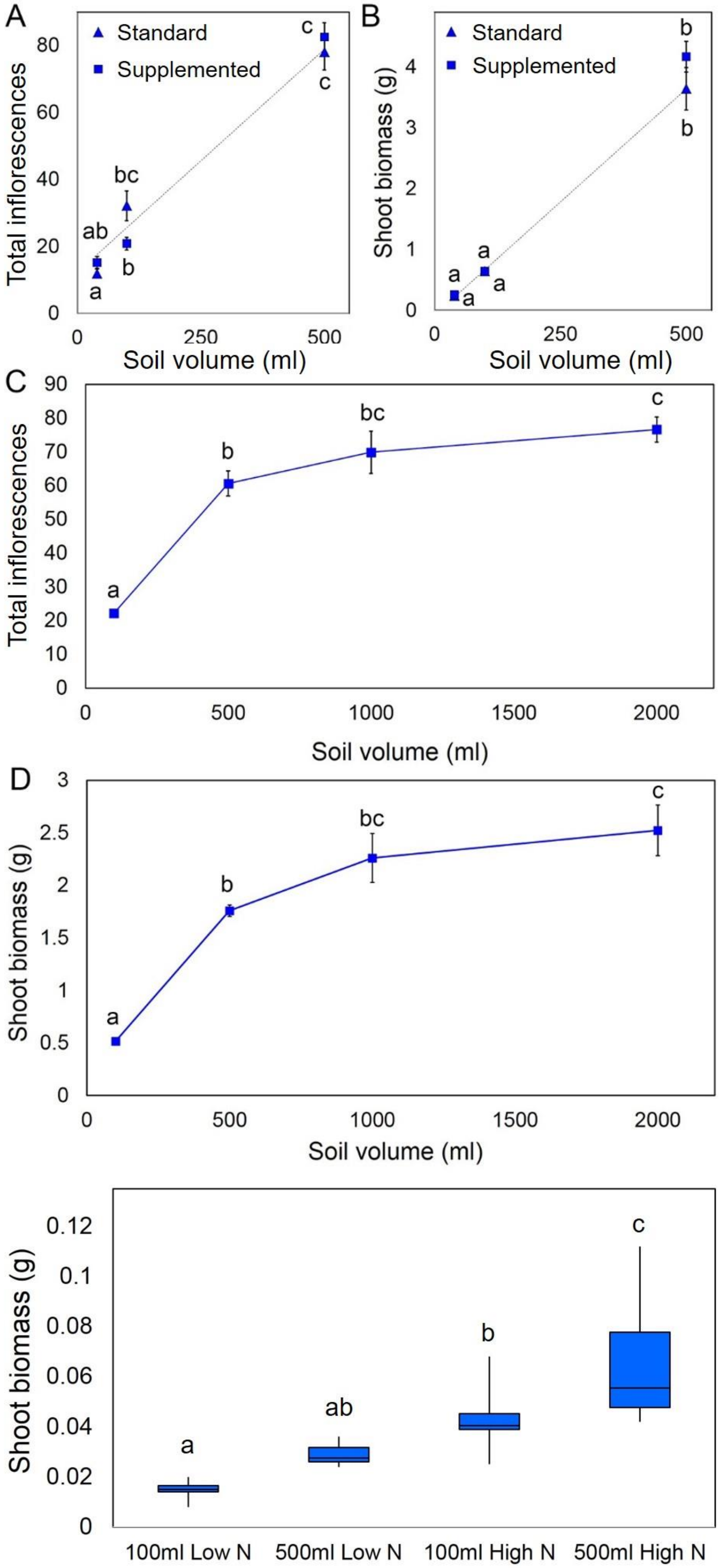
A
Standard
Supplemented
Total inflorescences
Soil volume (ml)
B
Shoot biomass (g)
C
D
100ml Low N
500ml Low N
100ml High N
500ml High N

Supplemental Figure S2: Substrate volume determines shoot growth in Arabidopsis

A,B) Graphs showing the relationship between soil volume and mean total branch number (A) and mean dry shoot biomass in grams (B) in Arabidopsis grown in 50, 100, and 500mL of soil, with ('Supplemented') or without ('Standard') additional nutrients. Error bars indicate s.e.m, n=10-12. Data points with the same letter are not statistically different to each other (A - Kruskal-Wallis test, F=54.94, df=5, $p>0.05$; B - ANOVA + Tukey HSD, F=118.3, df=5, $p>0.05$).

C) Graph showing mean total branch number in wild-type (Col-0) Arabidopsis grown on compost, in four pot sizes (100, 500, 1000 and 2000ml). Data points are means ± s.e.m. n=8-12. Bars with the same letter are not statistically different from each other (ANOVA+Tukey HSD, F=51.46, df=3, $p>0.05$).

D) Graph showing mean final dry shoot biomass in wild-type (Col-0) Arabidopsis grown on compost, in four pot sizes (100, 500, 1000 and 2000ml). Data points are means ± s.e.m. n=8-12. Bars with the same letter are not statistically different from each other (ANOVA+Tukey HSD, 39.69, df=3, $p>0.05$).

E) Box plots showing mean final dry shoot biomass in wild-type (Col-0) Arabidopsis grown on a sand/vermiculite mix, in two pot sizes (100 and 500ml) and supplemented with fertiliser containing either standard nitrate concentration (75µmol/week) or a low nitrate concentration (7.5µmol/week). Box represents interquartile range, and midline indicates the median. Whiskers indicate maximum and minimum. Data points are means ± s.e.m. n=10. Bars with the same letter are not statistically different from each other (ANOVA+Tukey HSD, F=24.82, df=3, $p>0.05$).

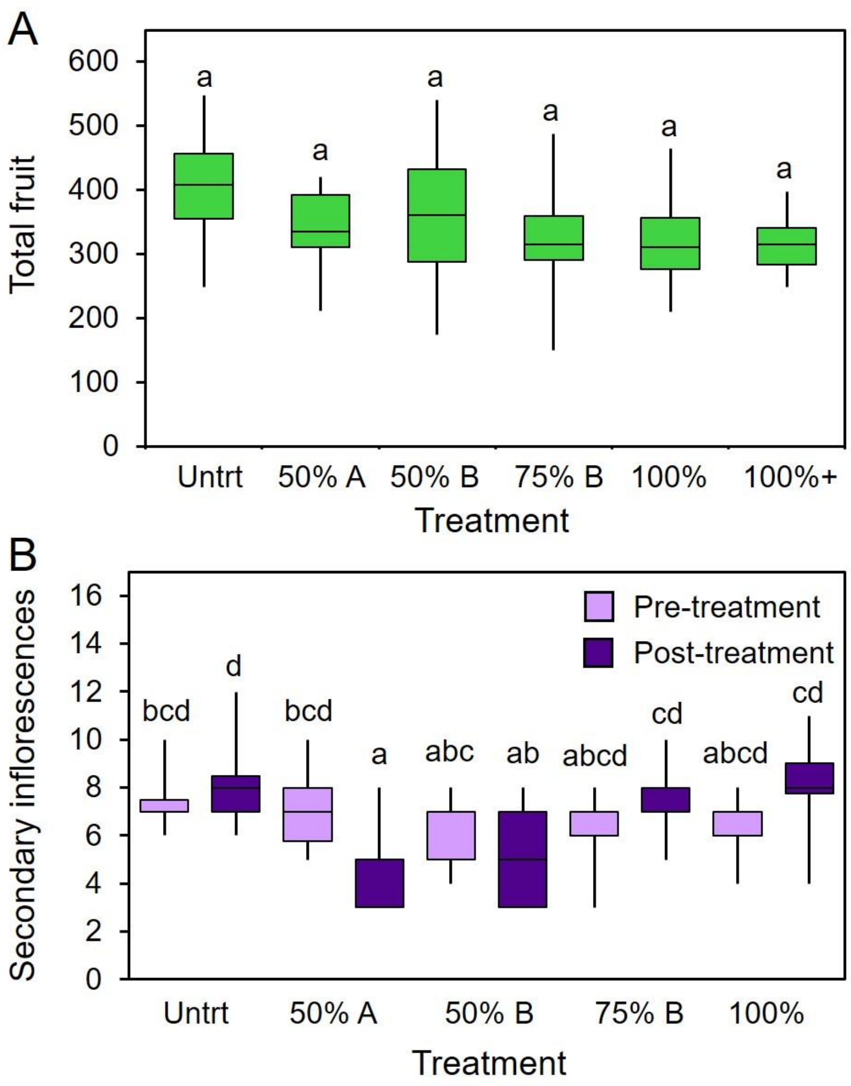

Supplemental Figure 3: Inflorescence and fruit number display homeostasis in Arabidopsis

A) Box plot of total fruits produced by Arabidopsis Col-0 under treated conditions. Secondary inflorescences were removed during flowering, and plants were allowed to recover; following the end of flowering, the total number of fruits on the plant was recorded. '50% A' had the apical 50% of existing secondary inflorescences removed, while '50% B' and '75% B' had the basal 50 or 75% of secondary inflorescences removed respectively. '100%' treated plants had all inflorescences removed, while leaving the primary inflorescence in-tact, while this was also removed in '100%+' treatments. Boxes indicate the interquartile range. The central line indicates the median, whiskers show minimum and maximum values. Bars with the same letter are not statistically different from each other (ANOVA + Tukey HSD, n=8-19, F=2.102, df=5, p>0.05).

B) Box plot of secondary inflorescences produced by Arabidopsis Col-0 plants. Secondary inflorescences (and any subtending higher order inflorescences) were removed from the plant with scissors following the end of flowering. Inflorescence numbers were recorded immediately prior to

treatment (pre-treatment, light boxes) and following a recovery period, when the plants were no longer flowering (post-treatment, dark boxes). ‘50% A’ had the apical 50% of existing secondary inflorescences removed, while ‘50% B’ and ‘75% B’ had the basal 50 or 75% of secondary inflorescences removed respectively. ‘100%’ treated plants had all inflorescences removed. Boxes indicate the interquartile range. The central line indicates the median, whiskers show minimum and maximum values. Bars with the same letter are not statistically different from each other (ANOVA + Tukey HSD, $n=9\text{-}13$, pre-treatment $F=1.960$, $df=4$; post-treatment $F=10.046$, $df=4$, $p>0.05$).

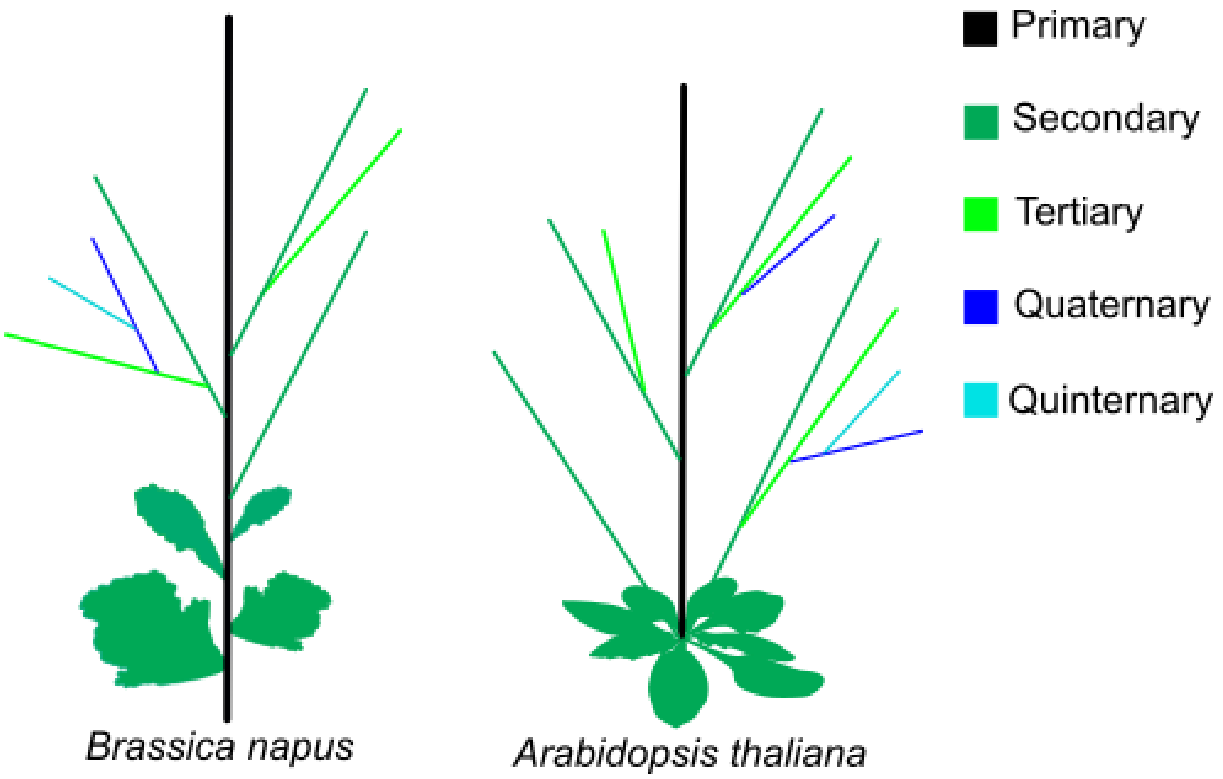

Supplemental Figure 4: inflorescence nomenclature in Arabidopsis and *B. napus*

Diagram indicating the inflorescence nomenclature for Arabidopsis and *B. napus*. The primary inflorescence (black) supports the leaves (*B. napus*) or arises from the vegetative rosette (Arabidopsis). Secondary inflorescences (dark green) arise from the primary inflorescence (both species), or the rosette leaves (Arabidopsis only). Tertiary inflorescences (light green) arise from secondaries, followed by quaternaries (dark blue) and quinternaries (light blue).
